# Associations between clinical and psychosocial factors and HbA1c in adult insulin pump users with type 1 diabetes

**DOI:** 10.1007/s00592-023-02081-4

**Published:** 2023-05-09

**Authors:** Signe Schmidt, Kristoffer Panduro Madsen, Ulrik Pedersen-Bjergaard, Karen Rytter, Eva Hommel, Bryan Cleal, Ingrid Willaing, Henrik Ullits Andersen, Kirsten Nørgaard

**Affiliations:** 1 Copenhagen University Hospital – Steno Diabetes Center Copenhagen, Borgmester Ib Juuls Vej 83, 2730 Herlev, Denmark; 2 Department of Clinical Pharmacology, Bispebjerg and Frederiksberg Hospital, Copenhagen, Denmark; 3 Danish Centre for Health Economics, University of Southern Denmark, Odense, Denmark; 4 Department of Endocrinology and Nephrology, Nordsjællands Hospital, Hillerød, Denmark; 5 Department of Clinical Medicine, University of Copenhagen, Copenhagen, Denmark; 6 Department of Public Health, University of Copenhagen, Copenhagen, Denmark

**Keywords:** Type 1 diabetes, Insulin pump, Questionnaire, Registries, HbA1c

## Abstract

**Aims:**

Many adults with type 1 diabetes do not achieve recommended glycemic goals despite intensive insulin therapy using insulin pumps. The aim of this study was to explore associations between clinical and psychosocial factors and HbA1c in insulin pump users to identify and prioritize areas for potential intervention.

**Methods:**

A questionnaire-based survey covering clinical and psychosocial aspects of life with type 1 diabetes was distributed to all adult (≥ 18 years) insulin pump users in the Capital Region of Denmark. Responses were combined with data from medical records and national registries. Associations with HbA1c were modeled using regression-based machine learning.

**Results:**

Of 1,591 invited individuals, 770 (48.4%) responded to the survey. Mean HbA1c among responders was 7.3% (56 mmol/mmol), and 35.6% had an HbA1c < 7.0% (53 mmol/mol). Six factors were significantly associated with HbA1c: diabetes duration (0.006% (0.1 mmol/mol) lower HbA1c per 1-year increase in diabetes duration); education (0.4% (4.3 mmol/mol) lower HbA1c with long higher education vs. primary school); insulin type (0.2% (2.2 mmol/mol) lower HbA1c with ultra-rapid-acting insulin vs. rapid-acting insulin); hypoglycemia awareness status (0.2% (2.2 mmol/mol) lower HbA1c with complete unawareness vs. full awareness); insulin device satisfaction (0.2% (2.7 mmol/mol) lower HbA1c per 1-point increase in Insulin Device Satisfaction Survey score); and diabetes distress (0.3% (3.1 mmol/mol) higher HbA1c per 1-point increase in Type 1 Diabetes Distress Scale score).

**Conclusions:**

This study identified several associations between clinical and psychosocial factors and HbA1c that may be considered when developing interventions targeted people with type 1 diabetes.

## Introduction

Too many people with type 1 diabetes have HbA1c above the recommended level [1–4]. In the US and in Europe, 40–60% and 5–15%, respectively, of the adult type 1 diabetes population use an insulin pump [2, 5], and on average this group of people have lower HbA1c than those who are using injection therapy[2, 6–9]. Nevertheless, for most insulin pump users, there is still a considerable gap between achieved and recommended glycemic levels.

Recently, advanced hybrid closed-loop insulin delivery systems were brought to market and this new category of insulin pumps has been shown to increase time in range and improve HbA1c compared with less advanced systems [10, 11]. However, the implementation of closed-loop systems is still at an early stage, not least due to the lack of reimbursement in some healthcare systems. While waiting for this new technology to reach more people with type 1 diabetes, it is worthwhile exploring how to improve outcomes of the less advanced insulin pumps currently in use, specifically how to decrease HbA1c, thereby reducing the risk of development and progression of micro- and macrovascular complications [12].

Despite a continuous increase in insulin pump use, knowledge about determinants of glycemic outcomes of the treatment method is limited in scope [13, 14]. A better understanding of what factors determine HbA1c—the modifiable as well as the non-modifiable—is important for people with diabetes and health care providers in directing and prioritizing their efforts and setting realistic expectations for the outcome of the therapy. Thus, to address this clinical need, we designed a questionnaire- and register-based multidimensional study to explore associations between clinical and psychosocial factors and HbA1c in adults with insulin pump-treated type 1 diabetes.

## Materials and methods

### Setting

In Denmark, diabetes care is free at the point of delivery through the publicly funded healthcare system. The healthcare system provides substantial subsidies for prescription medicines, including insulin. Insulin pumps are only available per prescription, and they are fully subsidized, including infusion sets and other disposables. All individuals with type 1 diabetes who use an insulin pump are trained and seen regularly in specialist diabetes clinics. The main indications for insulin pump therapy are not having achieved a HbA1c of 7.0% (53 mmol/mol) and recurring hypoglycemic events despite optimized intensive insulin injection therapy.

### Data collection

In June 2020, an online questionnaire-based survey was sent to all 1,591 adults (≥ 18 years) with type 1 diabetes treated with an insulin pump in the Capital Region of Denmark (1,423 were treated at Steno Diabetes Center Copenhagen and 168 at Nordsjællands Hospital Hillerød). This subgroup represented approximately 30% of all insulin pump-treated adults with type 1 diabetes in Denmark.

The questionnaire addressed several aspects of insulin pump therapy including device use and satisfaction, self-efficacy, psychosocial health, and general health behavior. For this study, we included only data derived from standardized questionnaire scales and excluded instruments which had not undergone psychometric validation. These instruments were (acronym and score range in parentheses): Insulin Device Satisfaction Survey (IDSS, 1-5) [15], Glucose Monitoring System Satisfaction Survey (GMSS, 1-5) [16], Hypoglycemia Fear Survey – short form (HFS-SF, 0-4) [17, 18], Type 1 Diabetes Distress Scale (T1DDS, 1-6) [19], WHO-5 Well-Being Index (WHO-5, 0-100) [20], and General Self-Efficacy Scale (GSES, 1-4) [21]. In addition, we included information from the questionnaire on insulin pump type, duration of insulin pump therapy, method of glucose monitoring, carbohydrate counting practices, physical activity level, smoking status, and alcohol consumption.

The full questionnaire, details about questionnaire development and data collection, and an analysis of differences between responders and non-responders are reported elsewhere [22]. Briefly, responders and non-responders differed slightly, the latter being younger (mean age 49 vs 33 years) and having higher HbA1c (7.3 vs. 7.6% (56 vs. 60 mmol/mol)) [22].

Questionnaire responses were supplemented with duration of type 1 diabetes, hypoglycemia awareness status according to Pedersen-Bjergaard criteria [23], and body mass index, which were manually retrieved from electronic medical records (EMR). Questionnaire and EMR data were subsequently linked to data from national registries by use of unique personal identifier numbers. Age, sex, marital status, number of children living at home and country of origin were obtained from the Population Registry; educational attainment was obtained from the Population Education Register [24]; and employment status and annual personal disposable income were obtained from the Income Statistics Register [25]. Diabetes-related complications were obtained from the Danish National Patient Registry [26]. This register captures diagnoses related to hospital-based in- and outpatient treatment; however, it does not include treatment received in non-hospital settings, e.g., treatment of severe hypoglycemia, unless the person is admitted to a hospital afterwards. Type of insulin (rapid-acting vs. ultra-rapid-acting insulin) used in the insulin pump was obtained from the Danish National Prescription Registry [27]. Finally, biochemical measures were obtained from the Danish National Laboratory Database [28]. For HbA1c, the latest available value within one year of the questionnaire distribution date was used.

The study was approved by the Danish Data Protection Agency (P-2019–812) and was presented to the Capital Region of Denmark’s Research Ethics Committee, which exempted it from further research ethical assessment (19,080,899). Nonetheless, the study was performed in accordance with the ethical standards laid down in the 1964 Declaration of Helsinki and its later amendments. Informed consent was obtained digitally before a participant could respond to the questionnaire.

### Statistical methods

We summarized sample characteristics with frequencies and shares (%) and medians with 25^th^ and 75th percentiles (25p/75p) or means with standard deviations (SD), depending on variable distributions. To explore which factors might explain heterogeneity in HbA1c, we stratified HbA1c level into those meeting vs. not meeting the recommended HbA1c (< vs. ≥ 7.0% (53 mmol/mol)) and analyzed crude differences with chi-squared test for categorical variables and 2-sided Student’s t-test or Wilcoxon rank-sum test for continuous variables.

Since clinical and psychosocial factors are often significantly correlated, disentangling the importance of one concept from that of another is difficult from a statistical point of view. Therefore, we categorized conceptually similar clinical and psychosocial factors into five groups of variables: 1) demographics and socioeconomics (age, sex, duration of type 1 diabetes, number of children living at home, country of origin, marital status, educational attainment, employment status, and annual personal disposable income), 2) diabetes management (insulin pump and glucose monitoring system types, duration of insulin pump therapy, type of insulin, carbohydrate counting practices, physical activity level, and smoking status), 3) diabetes health status (hypoglycemia awareness status, number of diabetes complications, and body mass index), 4) treatment satisfaction (IDSS and GMSS), and 5) psychosocial health (HFS-SF, T1DDS, WHO-5, and GSES). For each conceptual group, to identify factors or combinations of factors most strongly associated with HbA1c, we used a linear regression coupled with the least absolute shrinkage and selection operator (LASSO). LASSO is a machine-learning algorithm designed for model selection [29]. For a set of potentially explanatory variables, LASSO selects only those that independently add explanatory power to the model, thus avoiding issues of multicollinearity [30].

Specifically, we first estimated a ‘base’ model using HbA1c as the dependent variable with demographic and socioeconomic variables (group 1) entered as independent variables and used LASSO to select the most parsimonious model. Subsequently, we added each of the remaining four groups (group 2–5) of conceptually similar variables to the ‘base’ model in four separate linear regressions and used LASSO for model selection. All analyses used heteroskedastic-robust standard errors. Statistical analyses were performed in Stata 17 (StataCorp, TX, US).

## Results

In total, 770 individuals responded to the survey (response rate 48.4%). Median HbA1c for the study cohort was 7.3% (7.0–8.5%) (56 mmol/mol (50–62 mmol/mol)), of whom 35.6% had achieved the recommended target of < 7.0% (53 mmol/mol) (Table 1).

**Table 1:** Descriptive statistics of demographic and socioeconomic ‘base’ characteristics

	Total *N* = 770	HbA1c < 7.0% (53 mmol/mol) *N* = 274	HbA1c ≥ 7.0% (53 mmol/mol) *N* = 496	*p* value
Age, years	49.0 (36.0–60.0)	49.0 (34.0–60.0)	49.0 (37.0–60.0)	0.450 ^a^
Female (vs. male)	459 (59.7)	153 (55.8)	306 (61.8)	0.110 ^b^
Married (vs. not married)	439 (57.1)	167 (60.9)	272 (54.9)	0.110 ^b^
Number of children living at home	0.0 (0.0–1.0)	0.0 (0.0–1.0)	0.0 (0.0–1.0)	0.960 ^a^
Educational attainment				0.005 ^b^
Primary school	76 (9.9)	21 (7.7)	55 (11.1)	
High school or vocational school	288 (37.4)	106 (38.7)	182 (36.7)	
Short or medium higher education^c^	217 (28.2)	63 (23.0)	154 (31.0)	
Long higher education ^d^	189 (24.5)	84 (30.7)	105 (21.2)	
Employment status				0.840 ^b^
Employed	519 (67.5)	189 (69.2)	330 (66.5)	
Unemployed	52 (6.8)	17 (6.2)	35 (7.1)	
Retired	114 (14.8)	37 (13.6)	77 (15.5)	
Under education	84 (10.9)	30 (11.0)	54 (10.9)	
Annual disposable personal income (US dollar 1,000) ^e^	47.0 (33.8–62.0)	51.4 (35.8–67.0)	45.5 (32.9–59.9)	0.005 ^a^
Country of origin				0.580 ^b^
Denmark	731 (95.1)	261 (95.3)	470 (94.9)	
Nordics except Denmark ^f^	13 (1.7)	3 (1.1)	10 (2.0)	
Other countries	84 (10.9)	10 (3.6)	15 (3.0)	
Diabetes duration (years)	25.0 (18.0–40.0)	26.5 (18.0–41.0)	25.0 (18.0–38.5)	0.170 ^a^
HbA1c (%)	7.3 (6.7–7.7)	6.5 (6.3–6.8)	7.5 (7.3–8.0)	< 0.001 ^a^
HbA1c (mmol/mol)	56 (50–61)	48 (45–51)	59 (56–64)	< 0.001 ^a^

Data are presented as *n* (%) for categorical measures and mean (SD) or median (25/75 percentiles) for continuous measures. ^a^Wilcoxon rank-sum test. ^b^Pearson’s chi-squared test. ^c^Includes degrees from business academy and vocational college educations. ^d^Includes university degrees (bachelor, master, and doctorate degrees). ^e^The exchange rate between US dollar and Danish krone was 1 to 6.707 per June 1st, 2020. ^f^The Nordic countries include Denmark, Finland, Iceland, Norway, and Sweden

There were a number of significant differences between those with an HbA1c below and above 7.0% (53 mmol/mol) (Tables 1 and 2). Among those HbA1c below target there was a greater proportion with long higher education (30.7% vs. 21.2%) and annual disposable personal income was higher (51,425 vs. 45,535 USD) (Table 1). Body mass index and prevalence of smokers were lower in people who had achieved the HbA1c target, 25.8 (4.6) vs. 27.0 (4.8) kg/m^2^ and 8.2 vs 13.5%, respectively (Table 2). Further, ultra-rapid-acting insulin was more frequently used in insulin pumps of people with HbA1c < 7.0% (53 mmol/mol) (19.7% vs. 12.0%). Finally, people with the lowest HbA1c had higher levels of perceived self-efficacy (mean GSES score 32.0 vs. 30.6), less fear of hypoglycemia (mean HFS-SF score 0.8 vs. 1.0) and lower levels of diabetes distress (mean T1DDS score 1.8 vs. 2.0).

**Table 2:** Descriptive statistics of conceptually grouped variables

	Total	HbA1c < 7.0% (53 mmmol/mol)	HbA1c ≥ 7.0% (53 mmol/mol)	*p *value
*Diabetes management variables*				
Glucose monitoring system				0.200^a^
Blood glucose meter only	170 (22.3)	49 (18.1)	121 (24.7)	
isCGM/rtCGM (stand-alone)	268 (35.2)	101 (37.3)	167 (34.1)	
rtCGM (integrated; suspend functions)	261 (34.3)	96 (35.4)	165 (33.7)	
rtCGM (integrated; hybrid closed-loop)	62 (8.1)	25 (9.2)	37 (7.6)	
Insulin pump therapy duration (years)	9.0 (6.0–13.0)	9.0 (6.0–13.0)	10.0 (6.0–13.0)	0.780 ^b^
Ultra-rapid-acting (vs. rapid-acting) insulin	112 (14.7)	53 (19.7)	59 (12.0)	0.004 ^a^
Counts carbohydrates (vs. no counting)	693 (95.2)	247 (93.9)	446 (95.9)	0.230 ^a^
Physical activity (meets recommendations vs. not) ^c^	472 (66.4)	175 (68.1)	297 (65.4)	0.470 ^a^
Smoker (vs. non-smoker)	82 (11.6)	21 (8.2)	61 (13.5)	0.035 ^a^
*Diabetes health status variables*				
Hypoglycemia awareness status				0.092 ^a^
Normal awareness	449 (60.3)	146 (55.5)	303 (63.0)	
Impaired awareness	163 (21.9)	61 (23.2)	102 (21.2)	
Complete unawareness	132 (17.7)	56 (21.3)	76 (15.8)	
1 + diabetes complications (vs. 0 complications) ^d^	268 (34.8)	98 (35.8)	170 (34.3)	0.677 ^a^
Body mass index (kg/m^2^)	26.5 (4.8)	25.8 (4.6)	27.0 (4.8)	< 0.001 ^e^
*Treatment satisfaction variables*				
Insulin device Satisfaction survey score	4.3 (0.5)	4.4 (0.5)	4.3 (0.5)	0.190 ^e^
Glucose monitoring satisfaction Survey score	4.0 (0.6)	4.1 (0.6)	4.0 (0.6)	0.080 ^e^
*Psychosocial health variables*				
Hypoglycemia fear survey–short form score	0.9 (0.6)	0.8 (0.5)	1.0 (0.6)	< 0.001 ^e^
Type 1 Diabetes Distress Scale score	1.9 (0.6)	1.8 (0.5)	2.0 (0.6)	< 0.001 ^e^
WHO-5 Well-Being Index score	60.1 (20.1)	61.3 (19.0)	59.4 (20.7)	0.240 ^e^
General self-efficacy scale score	31 (6.0)	32.0 (6.0)	30.6 (6.0)	0.006 ^e^

Data are presented as *n* (%) for categorical measures and mean (SD) or median (25/75 percentiles) for continuous measures. ^a^Pearson’s chi-squared test. ^b^Wilcoxon rank-sum test. ^c^Meets WHO’s recommendation of 150–300 min of moderate-intensity exercise and/or 75–150 min of vigorous-intensity exercise per week. ^d^Diagnosis of retinopathy, nephropathy, neuropathy, and/or cardiovascular disease. ^e^Two-sample Student’s *t*-test

**Fig. 1:**
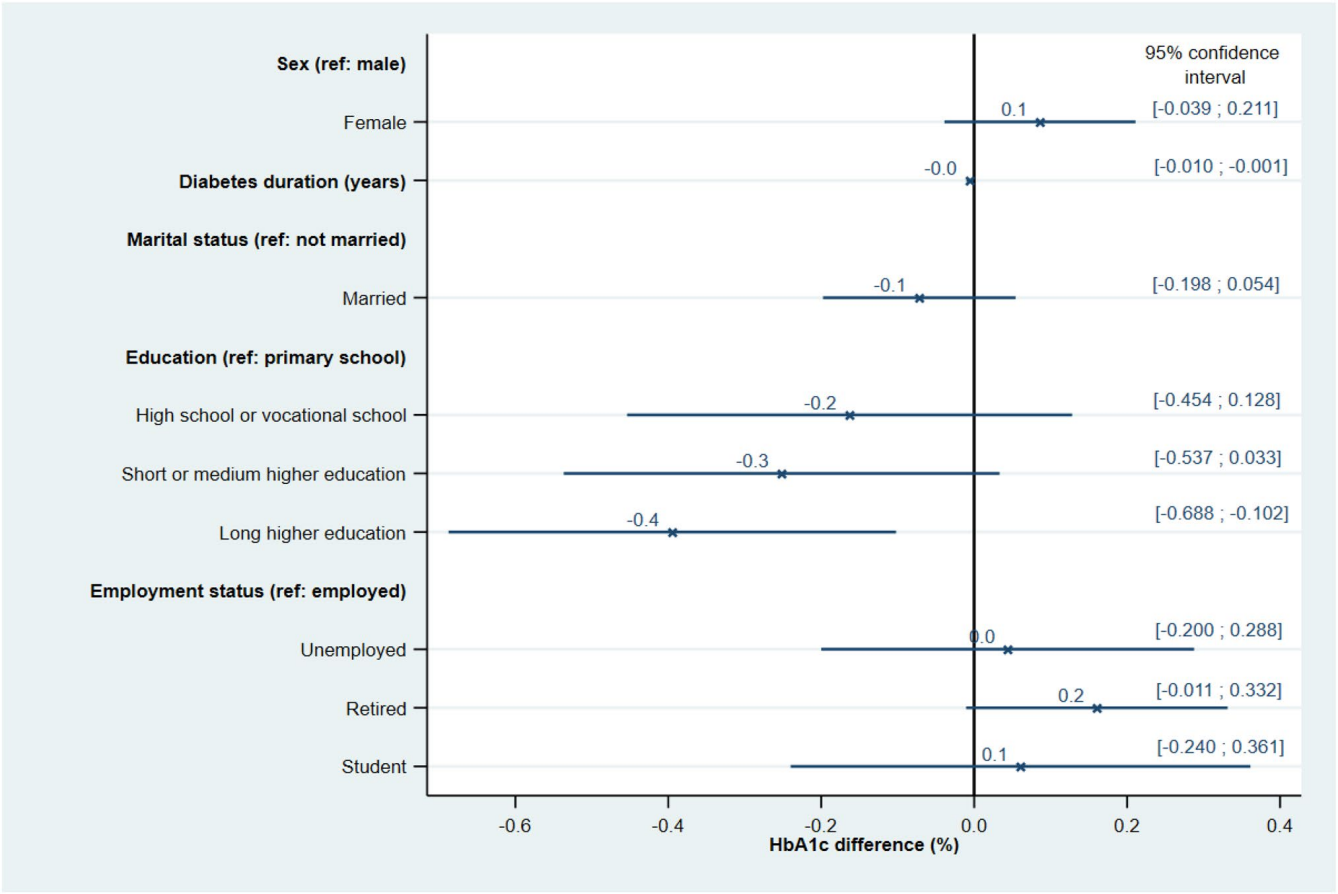
Associations between HbA1c and demographic and socioeconomic variables. LASSO-selected linear regression modeling the association between HbA1c and demographic and socioeconomic ‘base’ model variables

**Fig. 2:**
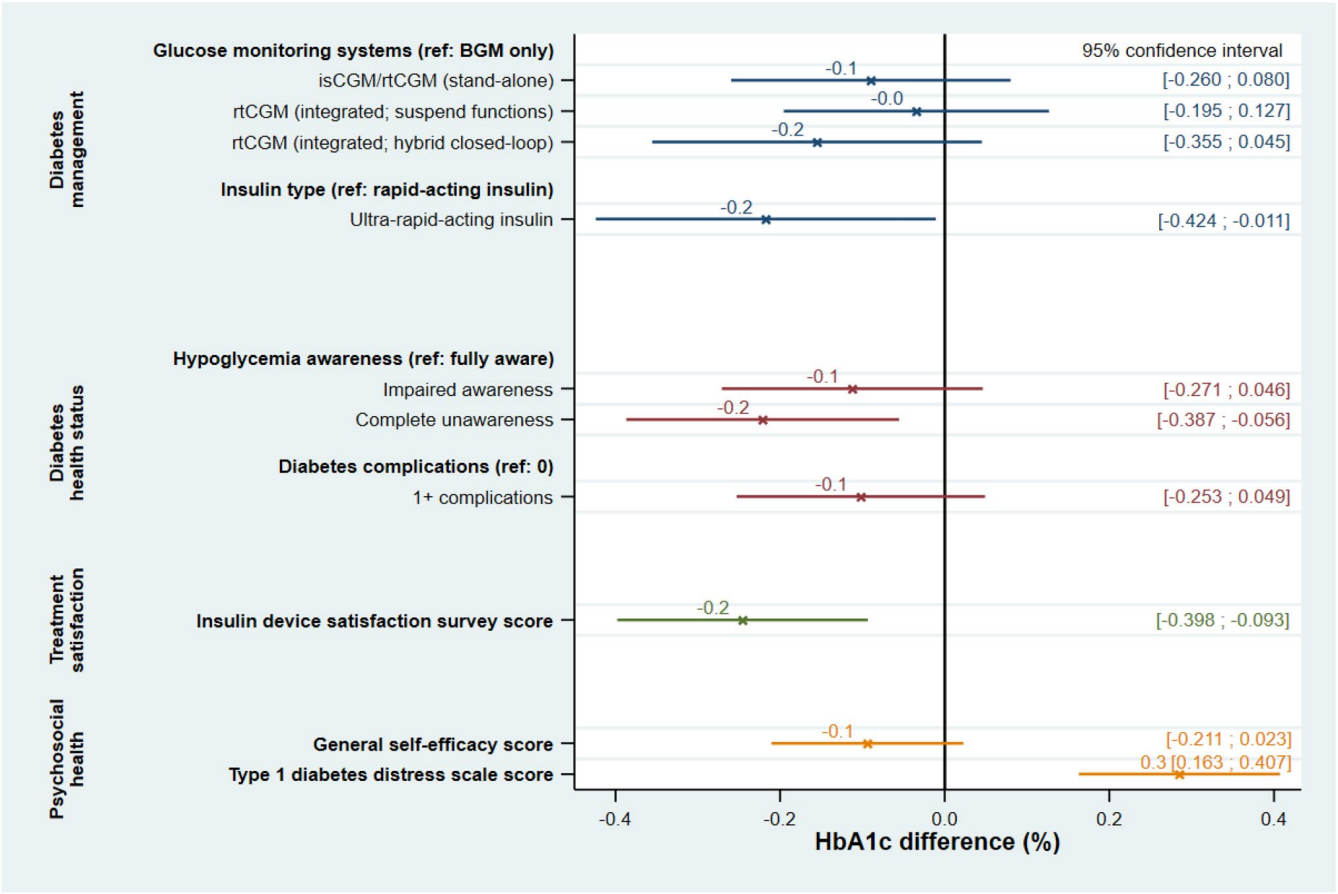
Associations between HbA1c and concept-grouped variables. The four subsections of the figure depict independent LASSO-selected linear regressions of the association between HbA1c and factors of, respectively, Diabetes management, Diabetes health status, Treatment satisfaction and Psychosocial health. Each regression was adjusted for sex, diabetes duration, marital status, educational attainment, and employment status as selected in the’base’ model

The LASSO-selected’base’ linear regression model that explained the most variance in HbA1c included sex, diabetes duration, marital status, educational attainment, and employment status. Among these variables, however, only two variables were significantly associated with HbA1c (Fig. 1): diabetes duration (0.006% (0.1 mmol/mol) lower HbA1c per 1-year increase in diabetes duration) and educational attainment (0.4% (4.3 mmol/mol) lower HbA1c with long higher education vs. primary school). By the subsequent combination of the’base’ model with each of the four concept-based groups (Fig. 2), another four variables were significantly associated with HbA1c: insulin type (0.2% (2.2 mmol/mol) lower HbA1c with ultra-rapid-acting insulin vs. rapid-acting insulin); hypoglycemia awareness status (0.2% (2.2 mmol/mol) lower HbA1c with complete unawareness vs. full awareness); insulin device satisfaction (0.2% (2.7 mmol/mol) lower HbA1c per 1-point increase in Insulin Device Satisfaction Survey score); and diabetes distress (0.3% (3.1 mmol/mol) higher HbA1c per 1-point increase in Type 1 Diabetes Distress Scale score).

## Discussion

In this cross-sectional survey of 770 adult insulin pump users with type 1 diabetes, which included self-reported and register-derived clinical and psychosocial measures, we found that longer diabetes duration, higher educational attainment, ultra-rapid insulin use, impaired hypoglycemia awareness, higher insulin device satisfaction, and lower diabetes distress were independently associated with lower HbA1c levels.

Educational attainment showed the strongest association with HbA1c among all variables included in this study. HbA1c was 0.4% (4.3 mmol/mol) lower in people with long higher education compared with people who left the education system after primary school. An inverse correlation between length of education and HbA1c in people with type 1 diabetes has been shown previously [31]. Likewise, an inverse correlation between personal income and HbA1c has been demonstrated by researchers before us [2]. However, when modeling associations of these variables with HbA1c, we found no independent explanatory power of personal income that was not already accounted for by educational attainment. For most adults, length of education is an unchangeable characteristic. Still, the observed HbA1c gap between people with different educational backgrounds may me narrowed by offering diabetes-specific education and support tailored to the educational level of the individual recipient.

Diabetes distress showed the second-strongest association with HbA1c. For each 1-point increase in diabetes distress score, HbA1c was associated with an increase of 0.3% (3.1 mmol/mol). Clinically significant diabetes distress, defined as a T1DDS score ≥ 2, was prevalent among insulin pump users in our study to a degree also seen in other type 1 diabetes populations [32, 33]. The high incidence of diabetes distress and its significant impact on HbA1c are disturbing. However, relative to, e.g., educational attainment, diabetes distress is a factor that could possibly be modified. Although psychosocial support is often not implemented in routine consultations, diabetes distress has been found responsive to interventions which can be incorporated into routine diabetes care [34, 35]. Thus, by prioritizing the emotional dimension of life with type 1 diabetes, a larger part of the population may be able to achieve their glycemic goal, and this underlines the importance of systematic screening and access to diabetes distress support [33]. Although our results and supporting literature show that more diabetes distress leads to higher HbA1c, this study cannot exclude that a bidirectional causal relation exists, i.e., that high HbA1c in itself may also contribute to diabetes distress [36].

Overall, insulin device satisfaction was high in the cohort. The treatment satisfaction model yielded a statistically significant negative association between insulin device satisfaction and HbA1c (− 0.2% (− 2.7 mmol/mol) per 1-point increase in IDSS score). The developers of the IDSS also found an inverse association between the two, but—contrary to us—with HbA1c as the explanatory variable and not an outcome per se [15]. Again, there are indications of a possible bidirectional association. It seems likely that an insulin pump user's satisfaction with glycemic outcomes of insulin pump therapy, e.g., HbA1c, is reflected in satisfaction with the insulin delivery device. However, it seems equally likely that satisfaction with the insulin pump reflects certain device characteristics such as ease of use which may have an impact on glycemic outcomes.

In the diabetes health status model, complete hypoglycemia unawareness was associated with an HbA1c of 0.2% (2.2 mmol/mol) lower than normal awareness status. This emphasizes that HbA1c should not be viewed as an independent treatment goal, but that the way in which the value is achieved should also be assessed. Low values should only be encouraged when they are achieved through maximizing time spent in the target glucose range and not when they are the result of substantial time spent in hypoglycemia. However, such evaluation of glucose values is only possible in people using continuous glucose monitoring (CGM) (intermittently scanned or real-time), leaving the remainder of the type 1 diabetes population subject to substandard care due to shortcomings of the surrogate marker, HbA1c. Although our results could indicate that people with hypoglycemia unawareness achieve lower HbA1c values than people who are aware, due to the cross-sectional study design we cannot exclude that it was low glucose values—and ultimately the low HbA1c—that made them unaware in the first place.

The diabetes management model revealed that use of ultra-rapid-acting insulin was associated with 0.2% (2.2 mmol/mol) lower HbA1c compared with use of rapid-acting insulin. This finding contrasts with the results of previous randomized controlled trials comparing fast-acting insulin aspart with insulin aspart in insulin pump users where no difference was found between the two insulin types in terms of HbA1c [37, 38]. Ongoing studies are further investigating the efficacy of ultra-rapid-acting insulins in insulin pumps. Based on the current study, we cannot conclude that type of insulin determines HbA1c. The associations found could also reflect that ultra-rapid-acting insulins—for various reasons—are more frequently prescribed to people with lower HbA1c values.

A final statistically significant finding was that HbA1c decreased with increasing diabetes duration. The clinical relevance of an absolute decrease in HbA1c of 0.006% (0.1 mmol/mol) per year since type 1 diabetes diagnosis is however questionable. There is no biological mechanism explaining the association, and although we may speculate about psychosocial mechanisms, we have no data and only sparse literature to support such hypotheses.

Interestingly, the diabetes management model revealed no significant association between HbA1c and insulin pump/glucose monitoring system type. This may be due to the needs-based selection process tied to initiating treatment with different systems in Denmark. That is, individuals who maintain acceptable glycemic outcomes with finger stick measurements are not prescribed sensor-based glucose monitoring.

Our study has several significant strengths: Firstly, we examined a wide range of variables that we hypothesized-based on scientific literature and our own clinical experience—could be associated with HbA1c in adult insulin pump users with type 1 diabetes. Other researchers have investigated factors associated with HbA1c in type 1 diabetes; however, often focus has been on relatively few factors per study, and study samples have often consisted of both people treated with insulin injections and insulin pumps [14, 39–41]. The current study is unique in that it combined multiple aspects of life with insulin pump-treated type 1 diabetes—self-reported and register-derived—in the same model whereby we were able to evaluate multicollinearity and estimate relative effect sizes. Secondly, a marked strength of our study was the high validity of data coming from national Danish registries. Thirdly, the study was conducted within a publicly funded healthcare setting in which people have equal access to diabetes services thereby reducing potential selection bias arising from heterogeneity in socioeconomic status.

Regardless of several study strengths, we are limited by the cross-sectional design to conclude on associations only, not causal relationships. As discussed above, the causal pathways for several of the identified associations are unclear and call for further exploration in prospective setups. In addition, our study is limited by the lack of CGM data from the 600 participants (78%) using is/rtCGM. We did not have access to these data because, currently, national registries do not capture data from diabetes devices and there is no integration between EMRs and device software. Inclusion of CGM data would have allowed us to make time in range the focus of our analysis, instead of the surrogate marker HbA1c, and further facilitated a nuanced analysis of other key CGM-derived measures such as, for instance, time below range. Finally, we acknowledge that our findings may be slightly biased as a consequence of the younger and those with higher HbA1c being underrepresented in our sample as demonstrated previously [22].

## Conclusions

In conclusion, this study identifies associations between clinical and psychosocial factors and HbA1c that may be considered when developing interventions for people with type 1 diabetes. Insulin type, hypoglycemia awareness status, insulin device satisfaction and levels of diabetes distress are potentially modifiable in the clinical setting. Whereas this is not the case for diabetes duration and educational attainment, awareness that heterogeneity in HbA1c exists over these factors may still be used to tailor interventions to the individual.
